# Resistance training and Down Syndrome: A narrative review on considerations for exercise prescription and safety

**DOI:** 10.3389/fphys.2022.948439

**Published:** 2022-09-27

**Authors:** Geiziane Leite Rodrigues Melo, Ivo Vieira de Sousa Neto, Eduardo Fernandes da Fonseca, Whitley Stone, Dahan da Cunha Nascimento

**Affiliations:** ^1^ Department of Physical Education, Catholic University of Brasília, Brasília, Brazil; ^2^ School of Physical Education and Sport of Ribeirão Preto, University of São Paulo (USP), Ribeirão Preto, São Paulo, Brazil; ^3^ Department of School of Kinesiology Recreation and Sport, Western Kentucky University, Bowling Green, FL, United States

**Keywords:** down syndrome, resistance training, intellectual disability, strength training, neuromuscular training, physical exercise program

## Abstract

The current manuscript reviews the literature on the health effects of resistance training (RT) for individuals with Down syndrome (DS), focusing on this training modality’s methodology, application, and safety. The literature has mentioned that early aging in this population is associated with loss of muscle strength, lower lean and bone mass, and increased obesity. It is necessary to propose non-pharmacological measures for prevention and health promotion. Thus, this review suggests a current research-based RT guide for individuals with DS. This review is divided into three sections: Section 2 briefly reviews DS and the effects on structural and functional decline and how exercise and physical activity can influence health aspects in this population; Section 3 summarizes the evidence for RT prescription; Section 4 briefly reviews the health and potential benefits of RT in individuals with DS. The findings from this review suggest that most individuals with DS should engage in moderate-intensity RT at least 2 days a week and perform RT on the major muscle groups and include balance training. The RT program should be modified and adapted according to individuals’ characteristics and limitations. RT promotes positive, health-related benefits such as increasing strength, improving body composition, improving functional capacity and balance, reducing inflammatory status and oxidative stress, and improving the immune system. The RT protocols summarized in this current review provide guidance, critical conclusions, and novel research settings, which could be useful to coaches, clinicians, and researchers to effectively design RT program for individuals with DS.

## Introduction

Approximately 1.5 billion people worldwide live with disabilities (PLWD); PLWD are less likely to meet the recommended physical activity guidelines (Ginis et al., 2021). Recognizing the disparity between PLWD and able-bodied populations, the World Health Organization (WHO) created the first set of recommendations for physical activity and sedentary behavior for this particular group (Bull et al., 2020). A common disability that affects activity behavior is Down syndrome (DS). DS is a chromosomal abnormality that occurs in a total or partial tripling of chromosome 21; intellectual disability (ID) represents a main characteristic of the condition (Bull, 2020). Individuals with DS are exposed to premature aging, high risk for congenital heart disease, atlantoaxial instability, Alzheimer’s disease (AD), thyroid disease, early muscle mass loss, and lower muscle strength (Barnhart and Connolly, 2007; Capone et al., 2018; Coelho-Junior et al., 2019; Foley and Killeen, 2019).

Life expectancy of individuals with DS is progressively increasing over time, from 25 years in 1983 to 60 years in 2020 (Bull, 2020; Guthold et al., 2020) due to improvements in medical technology, social care, and healthcare systems (Presson et al., 2013). Yet, the literature demonstrates that adults and older adults with DS are hospitalized more often than the general population and for a more extended period of time (Tenenbaum et al., 2012).

Compared to typically developing peers, people with DS have lower physical fitness and physical activity, with a higher prevalence of sedentary behavior regardless of age (Esposito et al., 2012; Suarez-Villadat et al., 2019; Oreskovic et al., 2020; Suarez-Villadat et al., 2021; Forseth et al., 2022). Adults with DS have increased adiposity, decreased bone mineral density, lean mass, and physical performance similar to or lower than older adults with sarcopenia (Coelho-Junior et al., 2019). These characteristics likely explain why people with DS exhibit lower levels of cardiovascular fitness, a higher prevalence of obesity, and lower muscular strength compared to the general population (Cowley et al., 2010; Fernhall et al., 2013; Bertapelli et al., 2016).

Individuals with DS consistently display reduced upper- and lower-body muscular strength compared to the general population (Pitetti et al., 1992; Cowley et al., 2011). The lower extremity weakness directly influences the ability to perform activities of daily living, such as walking, work-related tasks, going up and downstairs, or getting up and sitting on a chair (Shields and Taylor, 2010; Cowley et al., 2011; Lin and Wuang, 2012). This reduced muscular strength may be associated with attenuated lean body mass. The prevalence of sarcopenia is 14.3% in older adults with ID, with 12% of the sample having DS (Bastiaanse et al., 2012). Sarcopenia is positively associated with impaired mobility and inflammation (Bastiaanse et al., 2012). Lower lean body mass in adolescents with DS was related to reduced functional capacity and maximum oxygen consumption (a common metric for cardiorespiratory fitness) (González-Agüero et al., 2011a; González-Agüero et al., 2011b).

Resistance training (RT) is a non-pharmacological modality that potently attenuates muscle strength losses, improves body composition, and promotes functional capacity in young people and adults with DS (Shields et al., 2008; Cowley et al., 2010; Shields and Taylor, 2010; Cowley et al., 2011). While the number of studies investigating the effects of RT in individuals with DS has grown, the number of practitioners who use this training mode is still small (Sugimoto et al., 2016; Ginis et al., 2021). Cunningham et al. (2022) suggests that practitioners may not feel safe and are unsure about how to apply RT in individuals with an ID (Duplanty et al., 2014).

The purpose of this review was to provide scientific, evidence-based recommendations to health and fitness professionals regarding individualized prescription RT for individuals with DS, which has a high relevance in the exercise physiology and sports medicine. Our remarks may yield clinically useful information and help to effectively design RT interventions with safety accounted for. It is hoped that this review will inform directions for future research into RT monitoring and management, as well as stimulate a more open debate about the beneficial implications of RT on the long-term health in the DS context.

### Strength of evidence

The strength of evidence was based on and adapted from the American College of Sports Medicine (ACSM) position stand guideline (Chodzko-Zajko et al., 2009), Health Care Research and Quality (AHRQ) (West et al., 2002), and the evidence rating system of the National Heart Lung and Blood Institute (1998); this was used to evaluate published manuscripts and to determine the most appropriate RT studies with DS. The recommendations of this narrative review followed this classification:

1. Evidence level A (rich body of data): Randomized controlled trials that filled addressed the key questions of this study and population with randomization, blinding, interventions, outcomes, statistical analysis, results, discussion and funding with a large sample and results pointing in the same direction. Also displayed a substantial number of studies involving substantial numbers of participants.
2. Evidence level B (limited body of data): Limited number of randomized controlled trials or post-hoc of subgroup analysis of RCTs, or meta-analysis of RCTs that fulfilled all the key questions as study question, study population, randomization, blinding, interventions, outcomes, statistical analysis, results, discussion, and funding.
3. Evidence level C (nonrandomized trials and observational studies): Observational and uncontrolled studies.
4. Evidence level D (panel consensus judgment): Expert consensus opinion, case studies and consensus of panel members based on clinical experience or knowledge that does not meet the above-listed criteria.

## Down Syndrome

### Down Syndrome and effects on structural and functional decline

Due to the chromosomal anomaly, individuals with DS show a structural modification and functional decline in most physiological systems, even in the absence of disease (Roizen and Patterson, 2003; Bull, 2020). Among the functional declines, the loss of muscle strength and lower work capacity can be highlighted, which starts early and continues with advancing age (Cioni et al., 1994; Carmeli et al., 2002; Fernhall et al., 2013; Capone et al., 2018). However, the literature emphasizes that good physical fitness is essential for the proper performance of physical exercise because the individual becomes more tolerant to exercise and improves their functional abilities (Dodd and Shields, 2005; Sugimoto et al., 2016; Suarez-Villadat et al., 2021). Unfortunately, previous studies have shown that individuals with ID have low physical fitness which results in increased obesity, risk of falls, and chronic and neurodegenerative diseases (Rimmer et al., 2004; De Winter et al., 2009; Bayen et al., 2018; Suarez-Villadat et al., 2019; Kovačič et al., 2020). It was no surprise that lower baseline values of oxygen consumption (VO_2_) and a higher body mass to height ratio (BMI) were observed in individuals with DS across different age groups (Fernhall et al., 2013; Wee et al., 2015).

BMI might be a key factor in fitness levels and people with DS. When classified by BMI, those with normal body weight had better values of VO_2_ max and heart rate compared to those classified as obese (Wee et al., 2015). The change in body composition is another hallmark of individuals aging with DS; people with DS have lower lean mass and bone mineral density and a higher percentage of fat, regardless of sex, when compared to the general population (Angelopoulou et al., 2000; González-Agüero et al., 2011a). This has profound effects on the physical health and function of this population. Specific examples include the accumulation of body fat and its redistribution to central and visceral stores, increased risk of cardiometabolic and cardiovascular disease, and early sarcopenia (Bastiaanse et al., 2012; De Asua et al., 2014; Coelho-Junior et al., 2019; Kelly et al., 2019; Magge et al., 2019). A summary of these and other DS effects on structural and functional decline are provided in Table 1.

**TABLE 1:** Summary of Down Syndrome effects on structural and functional declines.

Variables	Typical Changes	Functional Significance
*Muscular function*		
*Motor performance and control* (Shields et al., 2010; Rigoldi et al., 2011)	Reaction time is increased in individuals with DS. The speed of movements like timed up and down stairs test or agility test is higher	It affects muscle tone, and consequently motor coordination. So, the movement will be less effective. Also, task learning time is longer
*Flexibility and joint range of motion* (Angelopoulou et al., 1999; Foley and Killeen, 2019)	Range of motion is greater in individuals with DS, especially in hip abduction. Excessive flexibility in hip abduction can occur due to hypotonia, hypoplasia of the pelvis, and shallow acetabulum	The maturation of joint structures and the neuromuscular system gradually reduces flexibility and range of motion with advancing age
*Cardiovascular function*		
*Vascular function* (Hu et al., 2013; Hilgenkamp et al., 2018)	Decreased vascular reserve and blunted arterial stiffness responses after maximal exercise are observed in individuals with DS. Also, they have reduced peripheral regulation of blood flow in response to sympathetic stimuli	Individuals with DS have smaller brachial diameters and shear rate. This will imply in the regulation of peripheral blood flow. In addition, autonomic dysfunction affects systemic regulation and peripheral blood flow, which directly impacts the vasoconstriction capacity of different systems, especially skeletal and cardiovascular muscle, during exercise
*Chronotropic incompetence* (Guerra et al., 2003; Fernhall et al., 2013)	Most individuals with DS exhibit chronotropic incompetence, regardless of age and sex. The maximum heart rate is 25–30 beats.min^−1^ lower when compared to people without disabilities	Chronotropic incompetence limits exercise intolerance
*Physical functional capacities*		
*Walking kinematics* (Beerse et al., 2019; Henderson et al., 2021)	Walking speed is slower. The stride length is shorter and wider. All spatio-temporal parameters have greater variability, except for step width and foot rotation angle	Implications for physical function (e.g., lower work capacity) and risk of falling
Isometric handgrip strength (Fernhall et al., 2013; Suarez-Villadat et al., 2019)	Maximum isometric strength is twice times lower regardless of age in individuals with DS.	Improving handgrip strength in this population with DS is necessary, because loss of muscle strength has been associated with premature mortality, lower functional capacity and metabolic morbidity (Bae et al., 2019)
*Anthropometry*		
*Height* (Agiovlasitis et al., 2020)	Short stature associated with this genetic syndrome are evident	Height significant decrease with older age
*Skeletal muscle index* (Coelho-Junior et al., 2019)	Higher SMI was positively correlated with a reduction of waist and hip circumference and fat mass	Loss of lean mass and bone mass reduces the SMI. Low SMI is a known predictor of morbidity and mortality (Janssen et al., 2002)
*Regional adiposity* (De Winter et al., 2012; De Asua et al., 2014)	Adults with DS have greater abdominal obesity than their non-DS peers. The prevalence of regional adiposity was 46% in adults with ID.	Individuals with DS with accumulation of visceral fat have higher rates of insulin resistance
*Metabolism*		
*Metabolic changes* (Fernhall et al., 2005; Flore et al., 2008; Phillips et al., 2013)	Resting metabolic rate and fat oxidation (during submaximal exercise) are lower in individuals with DS.	This might directly influence substrate utilization during exercise

DS, Down Syndrome; RT, resistance training; HR, heart rate; BMI, body mass index; SMI, skeletal muscle index; ID, intelectual disability.

### Down Syndrome and decreased physical activity

PLWD are less physically active than the general population, especially in low and middle-income countries (Bull et al., 2020; Ginis et al., 2021). A recent review found that by increasing the level of physical activity, there were significant improvements in the musculoskeletal and cardiovascular systems, mental and brain health among children, adolescents, and adults with disabilities (Ginis et al., 2021). Similar results were found in children with DS; 58% do not meet the 90 min per week minimum recommendations of daily physical activity (Shields et al., 2009) to promote benefits to adolescents with DS (Izquierdo-Gomez et al. (2015).

The rate of adults with ID meeting physical activity guidelines (13.5%) was less than half of the general population (30.8%) (Stancliffe and Anderson, 2017). Being physically active may be more difficult for individuals with DS due to genetic and phenotypic characteristics. Physical activity involves overcoming social, environmental, and physical barriers such as musculoskeletal disorders and cardiovascular changes that can influence physical and behavioral abilities (Mahy et al., 2010; Barr and Shields, 2011; Pitetti et al., 2013; Shields et al., 2017).

It may be difficult to motivate someone with DS to meet the 90-min activity threshold; fortunately, individuals with DS who performed 12 min/day of physical exercise displayed a higher level of physical fitness when compared with the control group and sedentary adolescents with DS (Izquierdo-Gomez et al., 2015). Physical activity is particularly important for people with DS due to the higher prevalence of mental health conditions (e.g., depression and anxiety) and risk of chronic health conditions such as stroke, diabetes, hypothyroidism, and AD (80% by the age of 65y) (Mccarron et al., 2017; Tsou et al., 2020).

**FIGURE 1:**
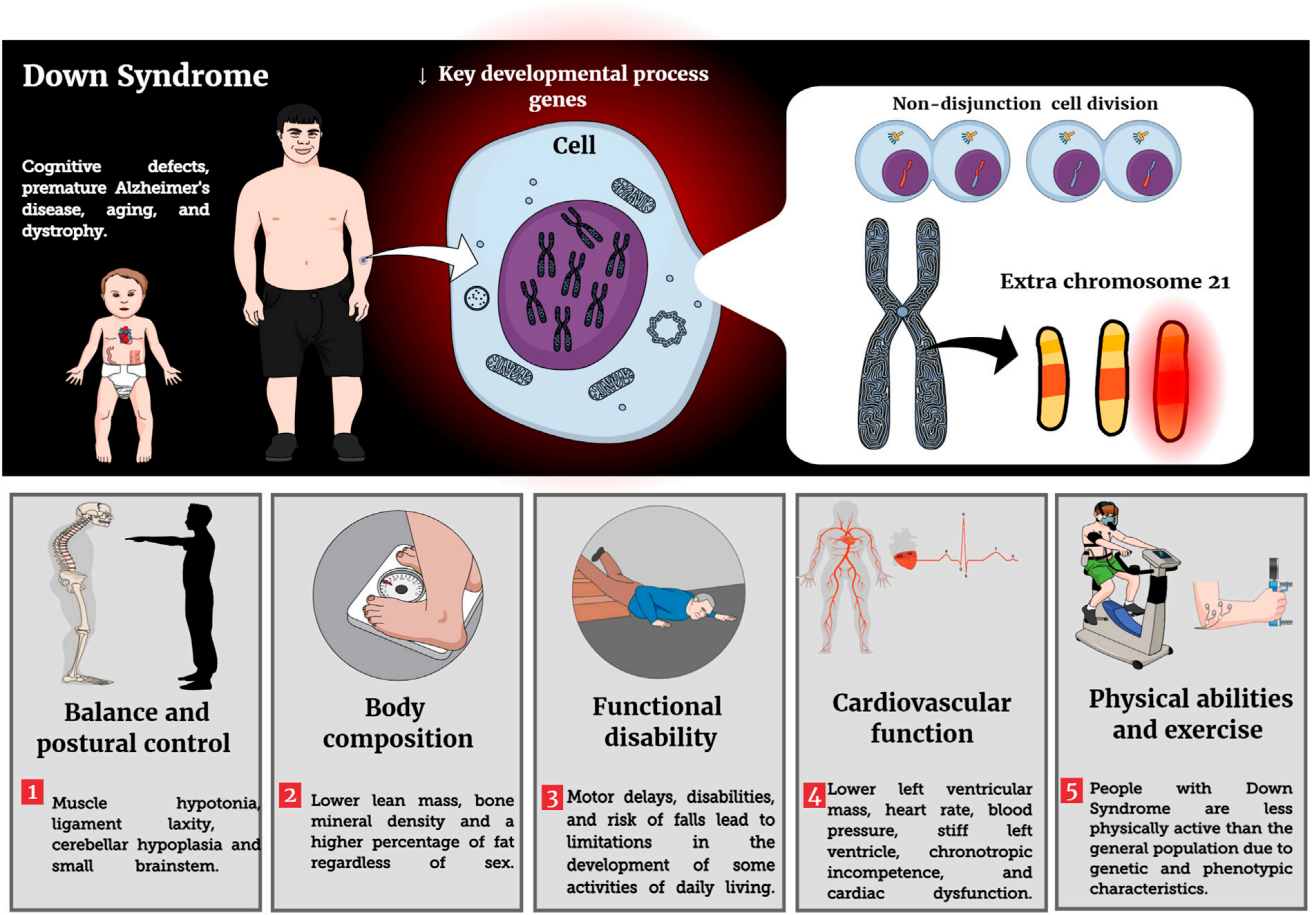
Effects of Down Syndrome on structural and functional decline.

Recent guidelines recommend that PLWD should perform physical activity as well as physical exercise (e.g., RT and balance exercise) at least twice a week, totaling 150 min of moderate physical activity (Naidoo et al., 2021). PLWD should consider adding RT to their regular activity profiles as it improves physical fitness, mental health, quality of life, increases autonomy and independence in activities of daily living, creates opportunities for affective and social relationships in the community, prevents chronic diseases (e.g., hypertension), improves mobility, balance and strengthens muscles and bones (Naidoo et al., 2021). To the authors’ knowledge, however, there is no summative work that has evaluated the literature on RT recommendations and its potential benefits in individuals with DS. Main impacts of DS condition on phenotypic characteristics are reported in Figure 1.

## Evidence for prescribing resistance training in individuals with Down Syndrome

The following section overviews RT programs in individuals with DS with specific focus toward the administration of safe, effective, and enjoyable schemes. Table 2 provide an overview of the recommendations for the application of RT in people with DS.

**TABLE 2:** Evidence statements and summary of recommendations for the individualized resistance training prescription in individuals with DS.

	Evidence-Based Recommendation	Evidence Category
Evidence for Prescribing Resistance Training		
Frequency	Large and small muscle groups should be trained on 2–3 times a week	B/C
Duration of training programs	The durations of training programs ranged from 6 to 21 weeks, but studies prescribed at 12 weeks were more frequent	B/C
Session Duration (min)	The structure followed warm-up, training and cool-down with a duration varying between 20 and 60 min	B/C
Exercise Load	1-Repetition maximum (1-RM)	C
	8 RM	B
	10 RM	C
	12 RM	B
Load	60–80% of the 1-RM (moderate to hard intensity) to improve muscular strength	B
	50–70% 1RM (moderate intensity) to improve muscular strength	B
	50% of the 1-RM (light intensity) to improve muscular endurance	C
	40–65% of the 8RM (very light to moderate intensity) for untrained to improve strength	B
	40–50% 8RM (very light to light intensity) to individuals with DS beginning exercise to improve strength	B
Type of exercise	Large and small muscle groups (arms and legs/bilateral)	B/C
	A variety of exercise equipment and/or body weight can be used to perform these exercises	B/C
Repetitions	6–12 repetitions are recommended to improved strength in most adults	B/C
	30 repetitions or repetitions to failure are recommended to improve muscular endurance	B
Sets	Two to three sets are the recommended for most adults to improve strength	B
	6 sets are effective in improving abdominal workout	B
Rest between sets (s)	Rest intervals of 90s between each set of repetitions are effective	B
Execution	Until concentric failure or when planned rep scheme is completed	B
Progression	A gradual progression of greater resistance, and/or more set, and/or increasing intensity is recommended	B/C
Evidence for health and potential benefits of RT		
Muscle strength	Individuals with DS, regardless of age, can substantially increase their strength after RT.	B/C
Body composition	RT improves lean mass and bone mass when participating in light to moderate intensity, while fat mass does not change	B/C
Muscle damage	Muscle damage does not change after RT using very light to moderate intensity protocol	B
Functional capacity	Improvements in functional capacity were reported after RT using protocols of moderate to higher intensity	B/C
Balance	Individuals with DS can substantially increase their static and dynamic balance after RT	B/C
Blood Pressure	RT can decrease or control BP in adults with DS; however, the precise RT prescription has not been established	C
Cardiorespiratory fitness	RT can improve functional exercise capacity; however, an exact exercise prescription has not yet been established	C
Oxidative stress	Improvements in oxidative stress have been demonstrated after RT in adults with DS.	B
Testosterone hormones and immunoglobulin A	RT can improve the immune system and the hormone testosterone	B
Inflammation	RT can decrease inflammation in the male adults with DS.	B
Mitochondrial dysfunction	Individuals with DS are more prone to fatigue and are intolerant to prolonged periods of exercise	C

DS, Down Syndrome; RM, repetition maximum; RT, Resistance training. Table evidence categories: A, randomized controlled trials (rich body of data); B, randomized controlled trials (limited body of data), C, nonrandomized trials, observational studies; D, panel consensus judgment.

### Exercise load

The RT programs presented different evaluations of the training intensity depending on the purpose of each study. Intensity was evaluated by a 1-repetition maximum (1RM) test (Weber and French, 1988; Shields et al., 2008; Shields and Taylor, 2010; Gupta et al., 2011; Rosety-Rodriguez et al., 2013; Shields et al., 2013; Dilek et al., 2018; Rosety-Rodriguez et al., 2021), eight repetitions maximum (8RM) (Davis and Sinning, 1987; Florentino Neto et al., 2010; Fornieles et al., 2014; Diaz et al., 2021), 10 repetitions maximum (10RM) (Cowley et al., 2011) or 12 repetitions maximum (12RM) (Florentino Neto et al., 2010; Seron et al., 2014; Seron et al., 2015; Seron et al., 2017). After application of the different RM tests, the intensity was expressed through the percentage of that workload which varied between 50 and 80% of 1RM (Weber and French, 1988; Shields et al., 2008; Gupta et al., 2011; Shields et al., 2013; Dilek et al., 2018; Rosety-Rodriguez et al., 2021), and 40–65% 8RM (Rosety-Rodriguez et al., 2013; Fornieles et al., 2014; Diaz et al., 2021). Despite presenting a range of different intensities, the summative results identified beneficial effects of RT in people with DS.

### Volume

The number of sets per exercise ranged from one to six (Davis and Sinning, 1987; Weber and French, 1988; Tsimaras and Fotiadou, 2004; Shields et al., 2008; Florentino Neto et al., 2010; Shields and Taylor, 2010; González-Agüero et al., 2011b; Cowley et al., 2011; Gupta et al., 2011; González-Agüero et al., 2012; Rosety-Rodriguez et al., 2013; Shields et al., 2013; Fornieles et al., 2014; Seron et al., 2014; Ghaeeni et al., 2015; Seron et al., 2015; Seron et al., 2017; Dilek et al., 2018; Diaz et al., 2021; Rosety-Rodriguez et al., 2021; Shin and Jeong, 2021); it varied according to the training periodization proposed by the objectives of the studies. However, most studies implemented two or three sets of each exercise (Davis and Sinning, 1987; Shields et al., 2008; Florentino Neto et al., 2010; Shields and Taylor, 2010; Cowley et al., 2011; Gupta et al., 2011; Rosety-Rodriguez et al., 2013; Shields et al., 2013; Fornieles et al., 2014; Seron et al., 2014; Seron et al., 2015; Seron et al., 2017; Dilek et al., 2018; Diaz et al., 2021; Rosety-Rodriguez et al., 2021; Shin and Jeong, 2021), with only one study using six sets (Ghaeeni et al., 2015). Most studies prescribed 6 to 12 repetitions (Davis and Sinning, 1987; Shields et al., 2008; Florentino Neto et al., 2010; Shields and Taylor, 2010; Cowley et al., 2011; Gupta et al., 2011; Rosety-Rodriguez et al., 2013; Shields et al., 2013; Fornieles et al., 2014; Seron et al., 2014; Seron et al., 2015; Seron et al., 2017; Diaz et al., 2021; Rosety-Rodriguez et al., 2021). However, the number of repetitions per set varied between 6 and 30. The prescription of 6–12 repetitions was related to the evaluation method of 1RM and maximum repetitions.

Some studies applied circuit exercises that used the time in each stage to perform maximum repetitions (Weber and French, 1988; Tsimaras and Fotiadou, 2004; González-Agüero et al., 2011b; González-Agüero et al., 2012; Shin and Jeong, 2021). Thus, the intensity and volume of training depended on the number of repetitions and sets, exercise order, and weekly frequency.

### Rest periods

The duration of the rest interval between sets ranged from 10 to 120 s (s), with most studies showing a rest interval of 90s (Rosety-Rodriguez et al., 2013; Fornieles et al., 2014; Diaz et al., 2021; Rosety-Rodriguez et al., 2021).

### Duration

Sessions were structured starting with a warm-up, main phase and return to calm/cool down. Time, or duration, of the sessions varied between 20 and 50 min, as suggested by the ACSM guidelines (Riebe et al., 2018). It should be noted that many studies failed to mention session duration (Davis and Sinning, 1987; Weber and French, 1988; Shields et al., 2008; Shields and Taylor, 2010; Cowley et al., 2011; Gupta et al., 2011; Rosety-Rodriguez et al., 2013; Shields et al., 2013; Fornieles et al., 2014; Diaz et al., 2021; Rosety-Rodriguez et al., 2021) as it is more typical to report sets and repetition schemes opposed to session durations.

### Frequency

Frequency varied between two and three times per week, with more leaning toward three sessions (Davis and Sinning, 1987; Weber and French, 1988; Tsimaras and Fotiadou, 2004; Florentino Neto et al., 2010; Gupta et al., 2011; Rosety-Rodriguez et al., 2013; Fornieles et al., 2014; Ghaeeni et al., 2015; Dilek et al., 2018; Diaz et al., 2021; Rosety-Rodriguez et al., 2021; Shin and Jeong, 2021). It has been shown that training twice per week leads to excellent results in muscle strength gains in upper and lower limbs, improvement in functional capacity, reduction of fat percentage, and increases in lean mass in a population with DS (Shields et al., 2008; Shields and Taylor, 2010; González-Agüero et al., 2011b; Cowley et al., 2011; González-Agüero et al., 2012; Shields et al., 2013).

### Duration of training programs

The duration of the programs ranged from 6 to 24 weeks, but most studies averaged at least 12 weeks (Tsimaras and Fotiadou, 2004; Florentino Neto et al., 2010; Rosety-Rodriguez et al., 2013; Fornieles et al., 2014; Seron et al., 2014; Seron et al., 2015; Seron et al., 2017; Diaz et al., 2021; Rosety-Rodriguez et al., 2021). Short-term intervention programs (lasting up to 6 weeks) had positive effects on upper limb strength and balance in adolescents with DS (Weber and French, 1988; Gupta et al., 2011). However, studies with a longer duration (21 and 24 weeks) resulted in additional positive adaptations to body composition in adolescents with DS (González-Agüero et al., 2011b; González-Agüero et al., 2012; Dilek et al., 2018).

### Types of exercise

Programs included in this review worked the upper and lower limbs’ muscles including both multi-joint and single joint exercises. These main exercises included the leg press, seated leg press, jumps, squat, deadlift; standing leg curl with ankle weights, one-sided stroke, seated row, lat pull-down, upright row, latissimus dorsi pull down, front pull-down, lateral rows, frontal rows, bench press, seated chest press, press-ups, press-ups on the wall, and abdominal exercises in their different variants.

The included investigations also implemented single-joint exercises for the biceps (bicep curl; arm curl and cable biceps curl), triceps (triceps extension; triceps curl, triceps pushdown; cable triceps extension; triceps French), shoulders (shoulder press; dumbbell front raise), calves (seated calf raise; calf raise; calf raises with ankle weights; heel lift), quadriceps (leg extension), hamstrings (leg curl), and hips (standing hip flex with ankle weights; flexors, abductors, extensors). In addition, three studies included plyometrics with different forms of execution (standing vertical jump, jump with run-in, drop jump, drop jump, and horizontal jump) for the lower limbs (Tsimaras and Fotiadou, 2004; González-Agüero et al., 2011b; González-Agüero et al., 2012; Dilek et al., 2018; Shin and Jeong, 2021).

In most studies, exercise on machines was chosen because it guides the movement and is safer in the initial phase of training (Weber and French, 1988; Shields et al., 2008; Shields and Taylor, 2010; Cowley et al., 2011; Rosety-Rodriguez et al., 2013; Shields et al., 2013; Fornieles et al., 2014; Seron et al., 2014; Seron et al., 2015; Seron et al., 2017; Diaz et al., 2021; Iversen et al., 2021; Rosety-Rodriguez et al., 2021). Most exercise prescriptions ranged from three to eleven exercises.

The literature recommends that exercises selected be simple and easy to understand for PLWD. In addition, exercise leaders should have a personalized follow-up to instruct and demonstrate each exercise in different ways so that the practitioner can verify if the individual can perform it correctly (Duplanty et al., 2014; Jacinto et al., 2021). Therefore, a period of familiarization with the exercises is essential for individuals with DS to learn the proper execution of the movement, eliminate the fear of using the equipment and perform the movement so that there are positive effects of the RT (Duplanty et al., 2014; Jacinto et al., 2021).

## Health and potential benefits of RT in individuals with Down Syndrome

Muscle strength. The literature has shown that RT is a safe, socially desirable, and viable option for individuals with DS across the lifespan (Davis and Sinning, 1987; Weber and French, 1988; Shields et al., 2008; Shields and Taylor, 2010; Shields et al., 2013; Sugimoto et al., 2016). RT improves muscle strength in individuals with DS represented by increases in maximal handgrip strength, isokinetic knee extension and flexion strength that had a magnitude of change of 19 and 27%, respectively, when compared to control group (Cowley et al., 2011; Rosety-Rodriguez et al., 2021).

Shields et al. (2013) reported a 21–30% increase in muscle strength among children and young adults with DS after 10 weeks of RT. These results were corroborated in adolescents (Shields and Taylor, 2010) and adults with DS (Shields et al., 2008; Cowley et al., 2011) who used similar RT programs (twice a week for 10 weeks). Adolescents experienced a 42% increase from baseline in lower limb muscle strength (Shields and Taylor, 2010) and adults gained 25% strength in the upper extremities (Shields et al., 2008). These positive findings after RT are witnessed in other studies noting improvements in muscle strength in the upper limbs (Davis and Sinning, 1987; Weber and French, 1988) and in the lower limbs with training (Weber and French, 1988). It is worth mentioning that RT is an important exercise modality that does more than simply impact muscular strength. Stronger extremities have been shown to improve functional capacity, and consequently, daily tasks and physical activities (Shields and Taylor, 2010). RT is suitable for individuals with DS as it is an activity that involves repetitive skills, similar to typical employment opportunities for PLWD.

### Body composition

Obesity is common in individuals with DS with approximately 50% of adults being obese. BMI and weight consistently increase between the age groups 18–29 and 30–39 and decrease after the age of 40, which may indicate the disease process (e.g., Alzheimer’s disease) in individuals with DS. Thus, it is necessary to be proactive in the prevention of muscle wasting and hypotonia that result in increased adiposity and decreased bone mineral density (Barnhart and Connolly, 2007; Presson et al., 2013; Capone et al., 2018; Coelho-Junior et al., 2019). Coelho-Junior et al. (2019) found that total lean mass is negatively correlated with age in individuals with DS, regardless of the muscle mass index.

González-Agüero et al. (2011b) found that 21 weeks of RT combined with plyometrics increased total upper and lower limbs lean mass and bone mass with no change in fat mass in children and adolescents with DS. These findings can be corroborated in the literature (Florentino Neto et al., 2010; Rosety-Rodriguez et al., 2013; Diaz et al., 2021). Interestingly, one study reported that a 12 week RT program was sufficient to reduce fat mass in adults with DS (Florentino Neto et al., 2010). This is not a universally supported finding as others failed to report a change in percentage body fat after 12 weeks of RT; alternatively, this ‘failure to change’ may have been a positive outcome as the control group increased the percentage of fat by 2.3% in the same period (Seron et al., 2014).

In addition, the active group experienced an increase in total bone mineral content (BMC), hip and lumbar region (6.7, 14.6 and 6.4%, respectively), when compared to the control group (González-Agüero et al., 2012). These promising results may have occurred due to the inclusion of plyometric jumps with RT, which was a strategy to improve BMC in young people with DS (González-Agüero et al., 2012). In general, there is a positive effect of plyometric exercise on osteogenesis (Vicente-Rodríguez, 2006). The connection may lie in the direct relationship between bone mass and total lean mass (Coelho-Junior et al., 2019; Kim et al., 2020). The practice of impact exercise such as running, plyometrics, and RT is associated calcium and vitamin D uptake to attenuate or prevent osteoporosis in individuals with DS (Angelopoulou et al., 2000; González-Agüero et al., 2012; Reza et al., 2013).

### Muscle damage

It is relevant to note that RT can induce muscle damage, especially with inappropriate prescription and unaccustomed exercise due to limited motor control, incorrect technique or a large amount of eccentric contractions. Excessive eccentric muscle actions can result in exercise-induced muscle damage or delayed onset muscle soreness in athletes, untrained and trained individuals (Clarkson and Hubal, 2002).

In this context, Diaz et al. (2021) evaluated the impact of RT on markers of muscle damage (creatine kinase activity, myoglobin concentration, and lactate dehydrogenase activity) in adults with DS. It was observed that there were no significant changes in the markers of muscle damage after 12 weeks or at any time during the intervention program. Another point worth mentioning is adherence to RT, which was 96% across 12 weeks. In addition, the participants did not present sports-related injuries or dropouts, as already demonstrated in the literature (Shields et al., 2008;Cowley et al., 2011; Shields et al., 2013; Diaz et al., 2021).

### Functional capacity

A number of assessments were used to evaluate functional tasks for lower-limb physical function in adolescents and adults with DS; the following tests were used: chair rise; ascend and descend 10 steps; walk speed; timed up and down stairs test, and timed get-up-and-go test (Shields et al., 2008; Shields and Taylor, 2010; Cowley et al., 2011; Rosety-Rodriguez et al., 2013). After performing the RT program (10 and 12 weeks), the participants showed a significant improvement in performance in all physical function scores of the lower limb muscles, except for the timed up and down stairs test and upper-body functional activities (e.g., grocery shelving task) (Shields et al., 2008; Shields and Taylor, 2010). Intensity and volume of training may have been insufficient to increase the functional capacity of both upper and lower limbs, despite having increased 21–30% of muscle strength (Shields et al., 2013).

Shields et al. (2013) reported that specificity of the training and duration of the RT program may have influenced the findings. For example, RT for 12 weeks improved the repetitive weighted box-stacking test (19.1 ± 3.0 vs. 23.3 ± 2.7 boxes/min; *p* = 0.0141) and pail-carry test scores were also significantly improved in the intervention group with DS (33.1 ± 5.7 m vs. 41.8 ± 6.0 m; *p* = 0.004) (Fornieles et al., 2014; Diaz et al., 2021). Though the duration might have played a factor in the divergent results, one could also argue training model adopted between studies could have influenced the findings. Shields et al. (2013) opted for the traditional RT model (e.g., performing less exercise in a higher amount of time) while Fornieles et al. (2014) and Diaz et al. (2021) increased intensity and volume every 2 weeks. The improvement of work task performance is of paramount importance for job prospects for adults with DS, as their activities in the workplace often require both physical and non-cognitive skills (Shields et al., 2008).

### Balance

Individuals with DS are deficient in postural balance, which can be explained by anatomical changes such as flat feet, muscle hypotonia, ligament laxity, cerebellar hypoplasia and small brainstems that generate disturbances in the balance regulation system or postural control (Lauteslager et al., 1998; Guzmán-Muñoz et al., 2017; Kim et al., 2017; Foley and Killeen, 2019; Shiohama et al., 2019). To perform movements of daily living or other motor tasks safely, it is essential to have postural control (Paillard, 2017). However, the literature has shown that the balance deficit in young people with DS can increase motor delays and disabilities, risk of falls and lead to limitations in the development of some activities of daily living (Lauteslager et al., 1998; Jung et al., 2017; Kim et al., 2017; Paillard, 2017; Maïano et al., 2019). There appear to be positive effects of different exercises (e.g., e-games, whole-body vibration, stretching, strength, and balance) on the static balance and dynamic balance of children and adolescents with DS (Maïano et al., 2019). Exercise as simple as walking on the treadmill and core strengthening improves static and dynamic balance in children with DS (Alsakhawi and Elshafey, 2019). These findings provide a powerful argument that people with DS should start and/or maintain regular resistance exercises.

Various RT programs were effective at improving balance. Gupta et al. (2011) reported a significant improvement in the Bruininks Osteresky Test of Motor Proficiency (BOTMP) balance subscale scores from 10.50 to 19.50 in the experimental group after strengthening the muscles of the pelvic girdle and lower limbs for 6 weeks (Gupta et al., 2011).

### Blood pressure

About 45–65% of people with DS have a congenital heart disease that affects the structure and function of the heart and vascular system (Capone et al., 2018). As a result, previous studies have shown that people with DS have chronotropic incompetence, lower heart rate (HR), altered cardiac autonomic function during exercise, lower blood pressure (BP) and resting heart rate when compared to individuals without DS (Fernhall and Otterstetter, 2003; Iellamo et al., 2005; Agiovlasitis et al., 2010; Fernhall et al., 2013; De Carvalho et al., 2015). According to De Carvalho et al. (2015) autonomic dysfunction occurs due to changes in sympathetic and parasympathetic tone that are associated with early morbidity and mortality. Hence, one study found that increased BP in adults with DS was associated with higher body mass index (Pucci et al., 2016).

The practice of physical exercise is essential to improve body composition and cardiovascular health. Seron et al. (2015) demonstrated that after 12 weeks of RT overweight adults with DS had a significant reduction in BP levels (systolic BP = -6.2 mmHg; diastolic BP = -4.8 mmHg; mean BP = -4.2 mmHg). These results are promising considering that individuals with DS have a higher incidence of cardiovascular risk factors such as low cardiorespiratory capacity, loss of muscle strength and obesity (Hu et al., 2013). Thus, numerous studies have demonstrated the beneficial effects of physical exercise that result in improving cardiopulmonary capacity, strength and body composition, and consequently improves cardiovascular parameters (González-Agüero et al., 2011b; Fernhall et al., 2013; Wee et al., 2015; Sugimoto et al., 2016; Jacinto et al., 2021).

### Cardiorespiratory fitness

Persons with DS have lower peak and submaximal exercise capacity as a result of numerous factors, among which are a lower level of physical fitness, alteration of autonomic cardiac function and catecholamine responses, prevalence of heart and pulmonary disease and chronotropic incompetence (Guerra et al., 2003; Iellamo et al., 2005; Fernhall et al., 2009; Mendonca G. V. et al., 2010). In addition, joint laxity and muscle hypotonia may influence poor exercise economy as it alters gait kinetics and kinematics (Mendonca G. et al., 2010).

On the other hand, a recent meta-analysis demonstrated that RT improved functional exercise capacity, endurance and peak exercise capacity in older adults with chronic obstructive pulmonary disease (Li et al., 2020). In addition to delaying the onset of neuromuscular fatigue in a submaximal aerobic test, RT improves strength and muscle functionality in aging adults (Emerson et al., 2015).

Seron et al. (2017) demonstrated that 12 weeks of RT increased pulmonary ventilation (VE) and total test time (TTT) in the treadmill test in young adults with DS. On the other hand, there was no significant difference in VO_2_peak after aerobic or RT interventions. However, the increase in maximum VE is of paramount importance for people with DS, as they present anatomical changes in the respiratory tract, such as a small nasal passage, which can hinder respiratory mechanics during exercise (Mendonca G. V. et al., 2010). In addition, the same study showed that increased time to exhaustion can increase cardiorespiratory endurance during exercise (Dodd and Shields, 2005).

### Oxidative stress

Oxidative stress can occur when there is an imbalance between generation and removal of reactive oxygen species (ROS) in the body. Unfortunately, oxidative stress is elevated from birth in people with DS (Jovanovic et al., 1998; Muchová et al., 2014).

A recent study in adults with DS showed that RT improved the antioxidant defense system and reduction in oxidative damage (Rosety-Rodriguez et al., 2021). After 12 weeks of RT (3x per week; 40–50% 8RM) active people with DS increased plasma total antioxidant status (TAS), erythrocyte glutathione reductase (GR) activity, and plasma levels of glutathione when compared control group with DS (Rosety-Rodriguez et al., 2021). The same study found a reduction in both markers of oxidative damage (malondialdehyde (MDA) and reduced carbonyl groups) in the intervention group. Despite the positive results of RT and oxidative damage, more work is needed to understand the effect of RT exercise on oxidative stress responses in different age groups, hence the potential role for RT to act as a stimulus for adaptation.

### Testosterone hormones and immunoglobulin A

People with DS have a higher prevalence of diseases related to the immune system when compared to the general population, due to low cellular and humoral immunity (Illouz et al., 2021). This occurs, in part, to the severe impairment of immunoglobulin (Ig) secretion in saliva (Chaushu et al., 2002). Thus, saliva is a predictor of susceptibility to respiratory tract infections (Chaushu et al., 2002).

Studies have demonstrated the positive effects of RT on the mucosal immune response as well as on salivary hormone levels in adults (Tsai et al., 2012; Schwanbeck et al., 2020). In more detail, Tsai et al. (2012) reported that RT promotes cumulative effects on IgA and cortisol responses in elite weightlifters. Likewise, RT with free weights significantly increased salivary testosterone and cortisol levels in male adults (Schwanbeck et al., 2020). Similar results were found in adults with DS after performing 12 weeks of RT (3x per week, 40–65% 8RM) such that there was an improvement in mucosal immunity response by increasing IgA concentration in the exercising group. Also, there was an increase in testosterone levels (Fornieles et al., 2014). However, no significant changes were observed in cortisol concentration after the RT program (Fornieles et al., 2014). It seems that short-term RT is a non-pharmacological alternative to improve the mucosal immune response as well as the salivary hormonal profile in individuals with DS (Fornieles et al., 2014). However, further studies are needed to confirm these results in different age groups.

### Inflammation

A meta-analysis evaluating inflammation reviewed 19 studies involving 957 children with DS and 541 controls. The investigation found significantly higher levels of Il-1β, TNF-α, IFN-γ and neopterin in individuals with DS (Zhang et al., 2017). However, the same study showed that circulating levels of interleukin (IL)-4, IL-6, IL-8 and IL-10 were not significantly different between those with DS and control groups (Zhang et al., 2017). Given these findings, RT has shown positive results to improve low-grade systemic inflammatory status after 12 weeks (3 days per week/40–65% 8RM). Male adults with DS experienced significantly decreased plasma levels of leptin, TNF- α and IL-6 while in the control group with DS had no change (Rosety-Rodriguez et al., 2013). The same study also highlights that there is a positive association between IL-6 and TNF-α and waist circumference (Rosety-Rodriguez et al., 2013).

The anti-inflammatory effects of RT may be mediated either by a reduction in visceral fat mass (with a subsequent decrease in adipokine release) or by an induction of an anti-inflammatory environment at each exercise session that will result in the production of IL-10, which counterbalances the pro-inflammatory state (Strasser et al., 2012). In addition, it should be noted that changes in body composition can directly influence adipokines, hence the importance of physical exercise in this physiological context (Ordonez et al., 2013).

Mitochondrial dysfunction. Mitochondrial dysfunction may contribute to premature aging, low levels of physical activity and AD in individuals with DS (Valenti et al., 2018). This is due primarily to the mitochondrial defect (in mitochondrial numbers or intrinsic mitochondrial activity) that leads to an imbalance between mitochondrial fusion and fission that results in inadequate mitochondrial energy production, impaired exercise tolerance, as well as intrinsic bioenergetic and metabolic dysfunction in skeletal muscle (Valenti et al., 2018; Pecze et al., 2020).

In this context, the literature has shown that after an acute session of maximal isokinetic strength testing in the quadriceps muscle, post-exercise phosphocreatine resynthesis (PCr) assessed by phosphorus magnetic resonance spectroscopy was 16% slower in adults with DS compared with non-DS, ID controls (Phillips et al., 2013). This result indicates a defect in mitochondrial respiratory function and alteration in biochemical/bioenergetic mechanisms in the skeletal muscle of this population (Phillips et al., 2013).

In addition, a recent study carried out in DS mice (model Ts65Dn) showed that 1 month of training (adapted aerobic exercise) did not improve body composition, however, they found that trisomy directly affects the metabolic response of skeletal muscle to exercise (Cisterna et al., 2022). The same study demonstrated that euploid mice have a better ability to restore energy storage (e.g., PCr) than trisomic mice. Unfortunately, it was observed that muscle regeneration in trisomic mice is attenuated due to reduced expansion of satellite cells, which maintain skeletal muscle, they are essential for muscle repair after exercise (Pawlikowski et al., 2018).

**FIGURE 2:**
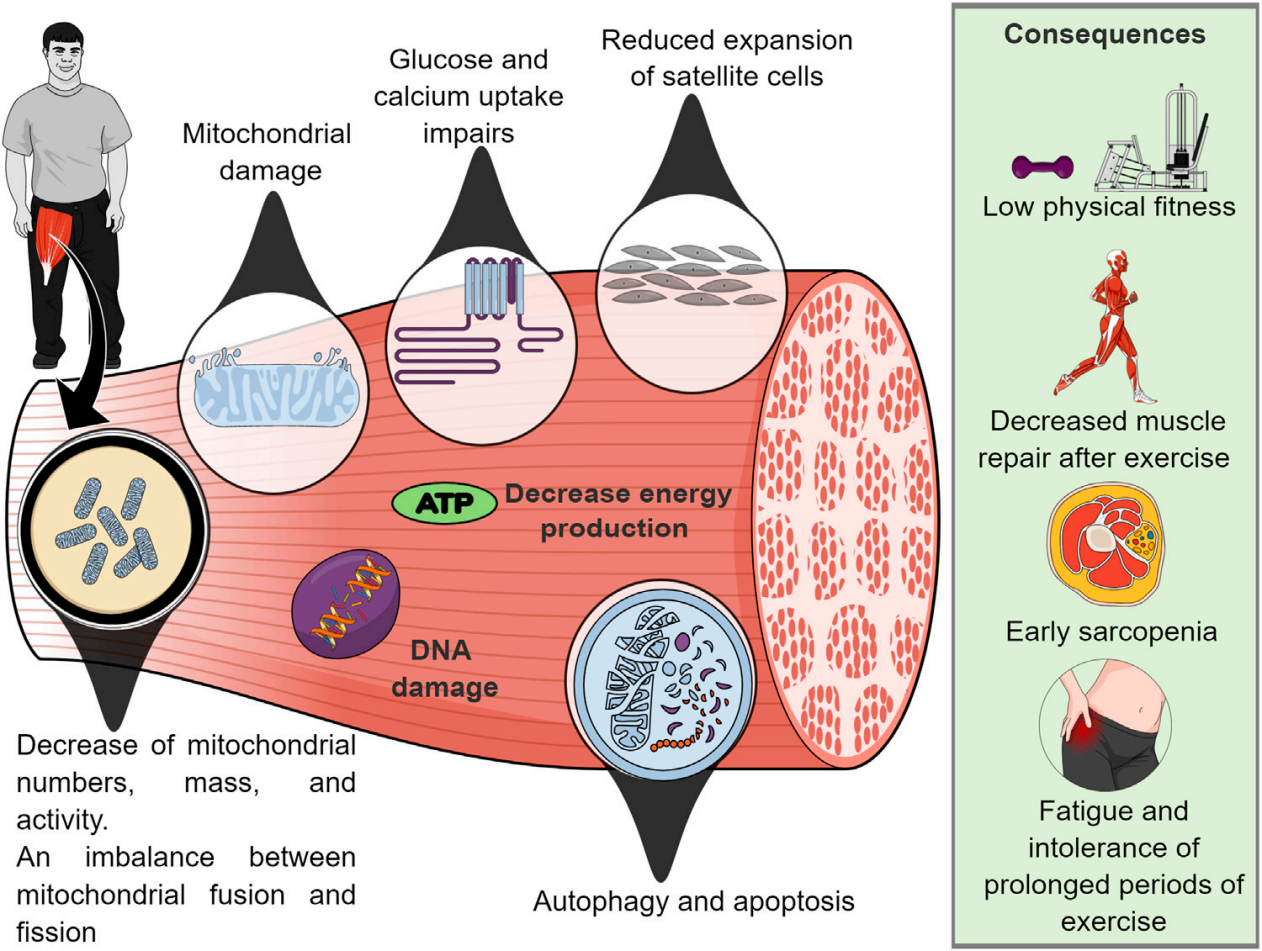
Role of mitochondrial dysfunction on skeletal muscle abnormalities.

Furthermore, Cowley et al. (2012) demonstrated that DS mice (model Ts65Dn) showed progressive skeletal muscle fatigue in repeated activation/recovery protocols. This contractile muscle weakness has been associated with alteration of different metabolic mechanisms (e.g. glucose and calcium uptake) as well as mitochondrial dysfunction (Hawli et al., 2009; Pecze et al., 2020). Thus, individuals with DS are more prone to fatigue and are intolerant to prolonged periods of exercise (Aguiar et al., 2008; Pecze et al., 2020). A summary of mitochondrial mechanisms that potentially mediate muscle dysfunction is reported in Figure 2.

## Discussion

**FIGURE 3:**
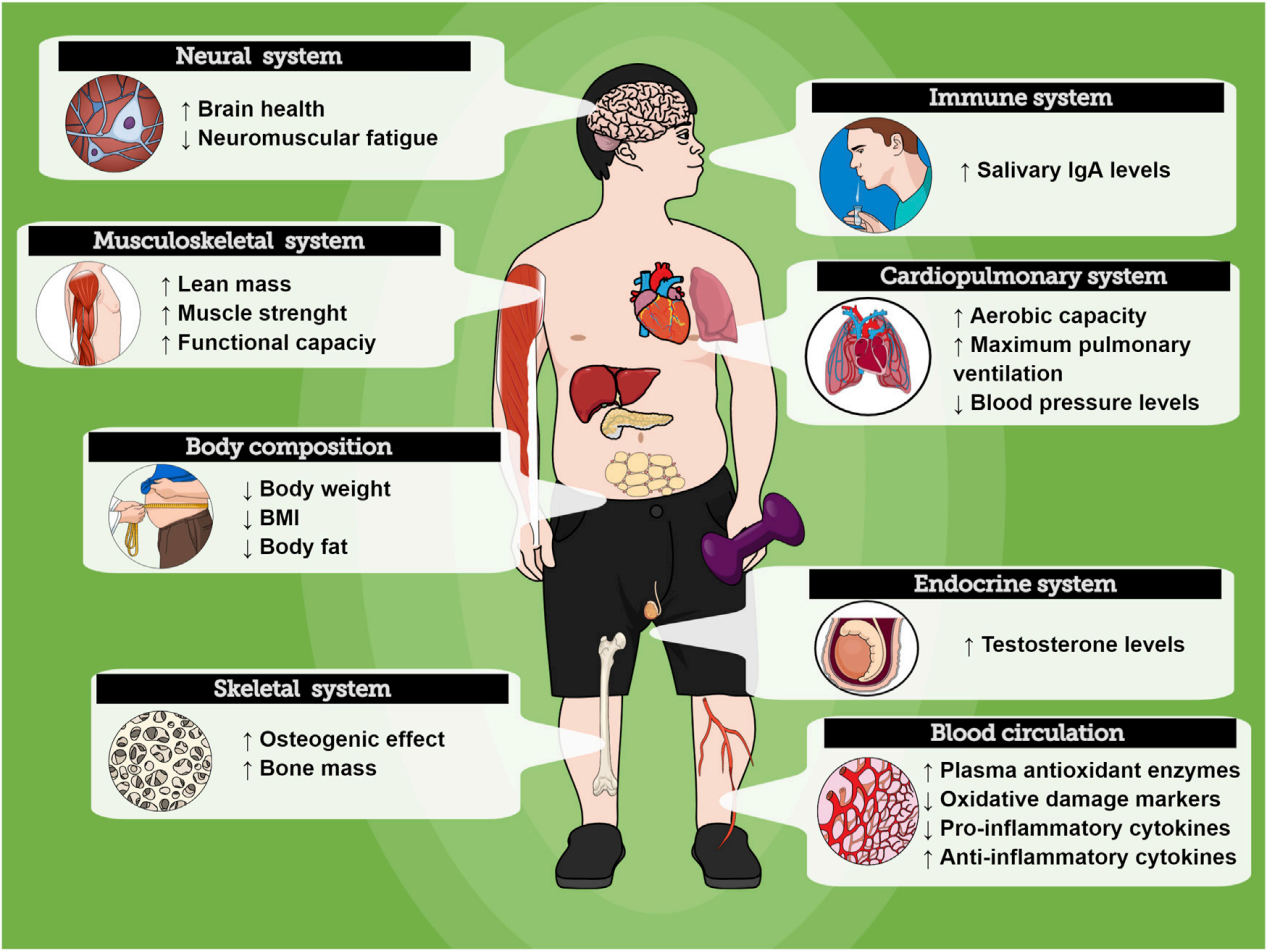
Overview of the RT effects on physiological systems in people with Down Syndrome. Exercise might promote positive health responses on different organs.

The evidence collected and reported in this review show promising health benefits of RT for individuals with DS. There is data to support that the RT increases muscle strength, and improves body composition, functional capacity, and balance, while reducing inflammatory status and oxidative stress and improving the immune system. Such effects can be important for health promotion, well-being, and longevity in individuals with DS. Furthermore, an emerging body of compelling data demonstrates that different RT protocols might induce beneficial effects on distinct tissues, organs, and physiological systems in individuals with DS (Figure 3). However, the overall methodological quality of the current literature is heterogenic in several key fields. To improve evidence quality, well-designed and standardized investigations with large samples are needed to establish the optimal parameters to exercise prescription.

The authors attempted to summarize the strength of the available scientific evidence on RT and individuals with DS noted in sections two and three of this narrative review. Although the narrative review does not use systematic research and analytical protocols, it is fundamental for understanding the mechanisms and suggesting possible gaps on the theme developed throughout this writing (West et al., 2002). Also, it is important to consider that a significant challenge arises in evaluating a body of knowledge comprising observational and randomized clinical trials (RCTs), and this important limitation should be considered in this review (West et al., 2002).

It was observed that there are still gaps in the literature regarding the effects of RT on cardiovascular responses, muscle damage, oxidative stress, inflammation, testosterone hormones and immunoglobulin A, considering that among these topics the authors found in the literature only one study, some of which were not RCTs (Rosety-Rodriguez et al., 2013; Fornieles et al., 2014; Seron et al., 2015; Diaz et al., 2021; Rosety-Rodriguez et al., 2021). In addition, most of these studies were in young adults with DS, except for the study on cardiovascular responses and RT (Seron et al., 2015).

Furthermore, future studies should evaluate the association between muscle mass/strength and chronic disease risk factors (inflammatory biomarkers, cholesterol level, insulin, systolic BP, diastolic BP and HR) in individuals with DS. We believe high priority should be placed on this issue because large cohorts showed that a decrease in muscle mass and strength is a predictor of chronic disease risk factors and all-cause mortality (Ruiz et al., 2008; Ruiz et al., 2009; Volaklis et al., 2015; Garcia-Hermoso et al., 2018; Jochem et al., 2019). This relevant topic might add new concepts and implications for public health in DS conditions.

Regardless of the advantages of RT, participation of people with DS in regular programs remains low in clinical practice, likely due to several aspects, such as time constraints, a high-perceived difficulty, and limited access to gyms, trained professionals, and equipment. Thus, the identification of exercise approaches that limit barriers to involvement may encourage engagement in RT and consequently enhance health outcomes are necessary. It was proposed that minimal doses of exercise, characterized by lower session volumes than in traditional RT guidelines, can improve muscle mass, strength, and functional capacity in younger and older people (Fyfe et al., 2022). Such minimal-dose strategies to RT have the capacity to reduce many barriers to exercise participation and may have beneficial implications for the feasibility and scalability in DS context.

Individuals with DS possess varying degrees of cognitive delays, from very mild to severe. Moreover, visual and hearing impairments vary greatly among individuals with DS, which can impact exercise learning. Associated with these factors, 70% of adults with DS develop AD-like neuropathology by 40 years of age and exercise adherence for persons with AD can be very challenging given their unique AD symptoms combinations (e.g., behavioral, and psychological symptoms of dementia) (Yu et al., 2017; Antonarakis et al., 2020). Unfortunately, there are no effective interventions to significantly delay or prevent the onset of AD, but people with DS need to be physically active as part of a healthy aging plan (Pape et al., 2021). On another hand, previous investigations have shown that physical exercise can prevent and promote improvements in the treatment of Alzheimer’s disease, as exercise improves modulation amyloid β turnover, inflammation, synthesis, and release of neurotrophins, and cerebral blood flow in elderly without DS (De La Rosa et al., 2020).

Optimal training prescription requires a person-centered approach using individualized strategies, with an appropriate balance of exercise stimulus, recovery, and optimal periodization to attain optimal physiological outcomes. From a clinical point of view, it seems to be important to start RT as early as possible to obtain the best physical performance, health outcomes, and quality of life. However, to aid the successful transfer to RT for individuals with DS, adherence, adhesion, and dropout reasons for an exercise program should be considered. Furthermore, one possibility that should not be ruled out is that individuals with DS must be supervised by a multidisciplinary team with long-term multifactor method (cognitive-behavioral therapies, individual diet, and medical intervention), in an attempt to comprehend the real impacts of RT on daily lives.

We propose that training acute variables (intensity, duration, volume, frequency, and interval) be controlled, addressed, and successfully integrated to create an effective intervention, regardless of the resistance exercise types. The optimal interplay of these variables can deeply potentiate training outcomes and requires attention, mainly in sedentary individuals with DS. We recommend the inclusion of progressive overload in order to maximize adaptions. However, there is a need for additional studies to understand the possible DS severity, age, and sex discrepancies in response to different RT protocols, besides the effects of interrupting exercise or detraining, and the impact of long-term training (>6 months) and lifelong RT, not just for a predetermined time.

Due to hypermobility of the joints, low muscle quality, and bone density related to DS, the prescription should be with higher caution than in younger healthy adults. Primary attention should be given to correct movement instruction and assertive verbal reports are essential. The selection of resistance exercises for individuals with DS should be based on specific needs, but that aim to improve static and dynamic balance, motor coordination, functional mobility, and consequently independence. Individuals that can tolerate bilateral closed kinetic chain exercises represent a great indication of intermuscular coordinated strength capacity (Fragala et al., 2019). Exercise training should reflect all major muscle groups in the upper and lower extremities with a priority for multijoint movements. For older adults with DS, a multicomponent exercise intervention program can be an alternative strategy for improving gait, balance, and strength, as well as reducing the falls rate (Fragala et al., 2019).

Given the complexity of biological systems in DS, the use of animal experimental and basic science might provide a meaningful understanding of the several adaptive mechanisms undergoing acute and chronic resistance exercise, particularly when ethical considerations make the use of human models unfeasible (Herault et al., 2017). Functional assessments, morphological, biochemical, and molecular approaches might aid understanding of the full picture of cellular adaptation in response to the RT program, besides the further discovery of potential therapeutic targets. A mechanistic framework of these responses could give valuable insights for therapeutic approaches to development and treatment guidance in humans, as well as clarify the main adverse and beneficial effects of RT in different physiological systems, tissues, and organs.

It is important for future research to verify the effects of RT in individuals with DS on variables highlighted in this narrative review with different age groups, especially after 40 years of age. People with DS are living longer lives and are exposed to early aging that may increase the risk of comorbidities and mortality in this population (Tenenbaum et al., 2012; Tsou et al., 2020). Finally, given the rapid development of research in this area, annual updates of this review are needed to keep pace with the latest findings regarding the RT prescription and safety for individuals with DS.

## Conclusion

The objective of this narrative review was to give an overview of the adaptations and benefits generated by RT with different intensities, volume, and application methods. The authors recommend the use of RT, as well as RT combined with different forms of exercise (aerobic, balance, plyometrics, and isometric), considering the volume and intensity, as well as the duration of training, type of exercise applied (e.g., machine exercise, bodyweight and/or free weights). Table 2 sets out the parameters by which professionals should use RT based on up-to-date and current research in the area. The RT protocols summarized in this current review provide guidance, critical conclusions, and novel research settings, which could be useful to coaches, clinicians, and researchers to effectively design RT program for individuals with DS.
